# Persistent Symptoms and Associated Risk Factors of COVID-19: A Cross-Sectional Study in Minia, Upper Egypt

**DOI:** 10.3390/healthcare13070699

**Published:** 2025-03-22

**Authors:** Asmaa Bassem, Amal Hussein, Mohamed Ahmed Sharawe Taha, Salah Mohamed El Sayed, Eman Mohamed Sadek, Hayam Ali AlRasheed, Mostafa M. Bahaa, Marwa Kamal

**Affiliations:** 1 Department of Clinical Pharmacy, Faculty of Pharmacy, Minia University, Minia 61519, Egypt; 2 Department of Pharmaceutics, Faculty of Pharmacy, Minia University, Minia 61519, Egypt; 3 Department of Internal Medicine and Nephrology, Faculty of Medicine, Minia University, Minia 61519, Egypt; 4 Department of Clinical Biochemistry, Taibah College of Medicine, Taibah University, Madinah 42353, Saudi Arabia; 5 Department of Pharmacy Practice, College of Pharmacy, Princess Nourah bint Abdulrahman University, Riyadh 11564, Saudi Arabia; 6 Pharmacy Practice Department, Faculty of Pharmacy, Horus University, New Damietta 44921, Egypt; mbahaa@horus.edu.eg; 7 Department of Clinical Pharmacy, Faculty of Pharmacy, Fayoum University, Fayoum 63514, Egypt

**Keywords:** long COVID-19 symptoms, risk factors, self-medication, cross-sectional study

## Abstract

**Background:** A significant number of COVID-19 survivors around the world have been reporting persistent symptoms following their recovery. Long COVID is recognized as a condition affecting not only the respiratory but also the gastrointestinal, cardiovascular, neurological, immune, and hematopoietic systems. **Objective:** This study aimed to describe persistent symptoms in COVID-19 survivors six months post-infection in Minia, Upper Egypt, and investigate associated risk factors. **Methods:** This observational cross-sectional study included 189 hospitalized and non-hospitalized patients previously diagnosed with COVID-19. Demographic data, symptom severity, comorbidities, and persistent symptoms were collected. A logistic regression analysis was used to identify risk factors associated with long COVID, with statistical significance set at *p* < 0.05. **Results:** In total, 68.8% of participants were women, and 83.5% of patients reported at least one ongoing symptom. The most self-reported symptoms were fatigue (73.5%) and myalgia (45.5%), followed by dyspnea (43.3%). Age was associated with an increased risk of developing long COVID (OR 1.028, 95% CI 1.003–1.054, *p* = 0.030). Patients who were hospitalized during the acute phase had more than twice the risks of having persistent symptoms (OR 2.384, 95% CI 1.055–5.387, *p* = 0.037). **Conclusions:** A substantial proportion of COVID-19 survivors in Minia, Upper Egypt, continues to experience persistent symptoms, primarily constitutional and neurological manifestations. Many patients reported self-medicating with unprescribed antibiotics, highlighting a need for public awareness regarding viral infections and the risks associated with improper antibiotic use.

## 1. Introduction

As of January 2025, more than 700 million cases of COVID-19 have been confirmed globally, with approximately 7 million deaths reported [1]. SARS-CoV-2 infection presents with a wide spectrum of symptoms, ranging from mild to life-threatening. Severe cases often lead to respiratory failure, necessitating mechanical ventilation and intensive care unit (ICU) admission. According to a meta-analysis conducted across nine countries, 9% of COVID-19 patients required invasive ventilation, while 2% needed extracorporeal membrane oxygenation (ECMO) [2]. Beyond respiratory complications, COVID-19 is associated with multi-organ involvement, including sepsis and multiple organ dysfunction syndrome (MODS) [3]. Extrapulmonary complications have been observed in the kidneys, heart, and liver, with acute kidney and cardiac injury occurring in approximately 20% of hospitalized patients in Wuhan, China [4].

Although the average recovery time from COVID-19 is estimated to be around two weeks, a substantial number of patients continue to experience persistent symptoms even after testing negative via polymerase chain reaction (PCR) testing. Long COVID or post-COVID-19 Condition (PCC) is a syndrome that typically manifests three months after the start of COVID-19. It is characterized by symptoms that last for at least two months and cannot be explained by another diagnosis, and it affects multiple organ systems. Early studies highlighted the burden of lingering symptoms. Many patients are being discharged from hospitals without a proper assessment of recovery. In Italy, a study conducted in April 2020 found that only 12.6% of a total of 143 patients were entirely free of any symptoms that relate to COVID-19, while 55% had three or more persistent symptoms. In total, at least one persistent symptom was reported in more than two-thirds of patients recovered from COVID-19, with fatigue and dyspnea being the two most commonly reported symptoms [5]. A study analyzing 1108 surveys found that nearly 29% of patients reported experiencing post-acute COVID-19. The most persistent symptoms identified were fatigue, as well as impairments in memory and concentration [6]. Furthermore, a study conducted in Assiut and Aswan University hospitals in Egypt reported that 23.9% of 439 hospitalized COVID-19 patients had non-specific neuropsychiatric symptoms such as headache, vertigo, and depression [7]. The evidence in the literature reflects our limited understanding of post-COVID-19 symptoms and highlights the importance of proper follow-up and the assessment of patients recovering from COVID-19, especially in Upper Egypt, as studies regarding long-term COVID-19 are still scarce, specifically in low-income nations years after the pandemic began. Moreover, because of the tendency of patients in Egypt to self-medicate without consulting a physician, the persistent symptoms and complications related to COVID-19 may be even more underreported when compared to other countries [8,9,10]. A study conducted in Tanta, Egypt, reported that 74.6% of students self-medicated, with antiviral medication accounting for 40.4%, antibiotics for 30.2%, and vitamin C and multi-vitamins for 25.6% [11]. We hypothesized that the behaviors and cultural attitudes toward healthcare of this unique demographic could affect patient outcome in comparison with other parts of the world.

Despite the growing body of evidence on long COVID, significant gaps remain, particularly in low- and middle-income regions like Upper Egypt, where research on post-COVID-19 symptoms is scarce. Additionally, self-medication is a widespread practice in Egypt, with many individuals using antibiotics without medical supervision [10]. This behavior may exacerbate post-COVID-19 complications, yet it remains understudied in the context of long COVID. Research conducted by Dooling et al. found that surveyed individuals in Egypt who used antibiotics without medical consultation contributed to antimicrobial resistance concerns. Most respondents thought antibiotic overuse contributes to resistance and reported “patient self-medication” as the biggest driver of overuse. Fifty physicians (21%) reported that they had prescribed antibiotics unnecessarily, citing patient over-the-counter access as the reason. Physicians < 40 years of age and those who treat adults were more likely to prescribe antibiotics for colds [12]. A study conducted by Barakat and Mohasseb reported that many Egyptians engage in self-medication with antibiotics, particularly for respiratory infections such as COVID-19 [8].

This study aims to investigate the prevalence and risk factors of long COVID in Minia, Egypt, with a focus on persistent symptoms and self-medication behaviors. Specifically, we seek to (1) identify the most common long COVID symptoms among survivors, (2) explore the association between symptom severity and pre-existing comorbidities, and (3) assess the extent of self-medication practices in this population. Addressing these gaps will contribute to a better understanding of long COVID in Egypt and inform future healthcare policies and patient management strategies.

## 2. Patients and Methods

### 2.1. Study Design

This is a cross-sectional observational survey study in which we collected information about any persistent or new symptoms after recovery from COVID-19. The average time after recovery from COVID-19 was 6 months at the time of patient interviews, and the median duration of symptoms was 14 days. A total of 402 individuals with a confirmed COVID-19 diagnosis were admitted to Minia University hospitals. A total of 189 patients agreed to participate and were included in the analysis based on convenience sampling. We collected various patient data including their medical history before COVID-19, treatments they received during their illness, and self-reported use of unprescribed antibiotics. We interviewed each patient using a set of questions about their symptoms, grading four main symptoms according to the severity on a 0–10 scale, where 0 = “absence of this symptom” and 10 = “this symptom is very severe”. Graded symptoms were fatigue, dyspnea, cough, and insomnia. Other symptoms were included in the questionnaire according to their presence or absence. To exclude other possible causes, information about the existence or any symptoms before the confirmation of their COVID-19 diagnosis was obtained.

### 2.2. Setting

This study was performed in Minia, Upper Egypt, between January 2021 and October 2021.

### 2.3. Ethical Approval

All procedures were conducted in accordance with the ethical guidelines outlined in the Declaration of Helsinki and were approved by the ethics committee of Minia, the Faculty of Pharmacy, Minia, Egypt (IRB; Code No. HV11/2020). Prior to participation, all individuals were provided with a comprehensive explanation of this study’s objectives, procedures, potential risks, and benefits. Written informed consent was obtained from each participant before data collection, ensuring that participation was entirely voluntary. For illiterate participants, verbal informed consent was obtained in the presence of a witness, following ethical standards. To ensure participant confidentiality, each participant was assigned a unique identification (ID) number, and all data were recorded and analyzed using these IDs instead of real names or any personally identifiable information. To protect participants’ confidentiality, all collected data were anonymized and stored securely. Identifiable information was removed from the dataset before analysis, and responses were coded to prevent any linkage to individual identities. Additionally, access to the data were restricted to authorized researchers only, and findings were reported in aggregate form to maintain privacy.

### 2.4. Inclusion Criteria

Patients were included in this study if they tested positive for COVID-19 at the time of their illness (positive COVID-19 polymerase chain reaction [PCR] test) or if laboratory, clinical, and radiological criteria suggested the infection with COVID-19, and the severity of their symptoms ranged from asymptomatic to severe. Both hospitalized and non-hospitalized patients were included.

### 2.5. Exclusion Criteria

Patients excluded were frail, at the end of their life, or had severe cognitive dysfunction, as well as patients < 18 years of age.

### 2.6. Bias

The questionnaire was completed with the aid of well-trained interviewers. No interviewers had an association with or worked at Minia University Hospitals at the time of patient interviews. To minimize recall bias regarding the use of self-prescribed antibiotics by elderly patients, we cross-examined the information given by the patients with a close family member for confirmation. Symptoms present before the onset of COVID-19 or that were related to other possible underlying conditions were excluded.

### 2.7. Sample Size Calculation

The sample size of 189 participants was determined based on feasibility and data availability, ensuring adequate representation of both hospitalized and non-hospitalized COVID-19 survivors in Minia, Egypt. Additionally, this sample size is comparable to similar studies investigating long COVID prevalence in other settings [13,14,15].

### 2.8. Missing Data

In our study, we handled missing data using a complete-case analysis approach, where only participants with fully available data for the key variables were included in the final analysis. In our study, participants with missing data regarding their diagnosis, hospitalization, or severity of the disease were excluded from the analysis to avoid potential biases.

### 2.9. Statistical Methods

Demographic information, medical history, and illness-related data (place of quarantine, symptom duration, and symptom severity) were analyzed using descriptive statistics. Categorical variables were expressed as frequencies and percentages, while continuous variables were presented as means and standard deviations. Simple logistic regression was performed to identify factors associated with the development of long COVID syndrome. Each potential predictor variable was analyzed individually in separate logistic regression models with long COVID syndrome (presence/absence) as the binary dependent variable. The following variables were included as potential predictors: age (continuous), presence of chronic conditions (yes/no), hospitalization (yes/no), and severity of symptoms (mild/moderate/severe). The results are presented as odds ratios (ORs), 95% confidence intervals (CIs), and *p*-values. The statistical significance level was set at *p* < 0.05. SPSS software was utilized for statistical analysis of the results.

Demographic and clinical data for all patients were age, gender, severity of symptoms of the acute phase of the infection, duration of symptoms, site of isolation, and the type and number of concurrent medical conditions (cardiovascular diseases, diabetes mellitus, stroke, chronic obstructive pulmonary disease (COPD), and asthma). Smoking status was also reported. Information about the body mass index (BMI) and the amount of alcohol consumption were not available. Data related to the main long COVID-19 symptoms were shortness of breath (SOB), cough, fatigue, and insomnia. Possible neurological symptoms were myalgia, headache, confusion, depression/anxiety, anosmia, ageusia, and deafness. Other symptoms included chest discomfort, loss of appetite, GIT symptoms, and edema. Drug history compromised the use of analgesics, antibiotics, corticosteroids, anticoagulants, and antivirals. Data about the length of hospitalization stay were collected.

## 3. Results

### 3.1. Demographic Data

Table 1 below shows the demographic data and clinical characteristics of the participants of this study.

### 3.2. Descriptive Data

**Figure 1:**
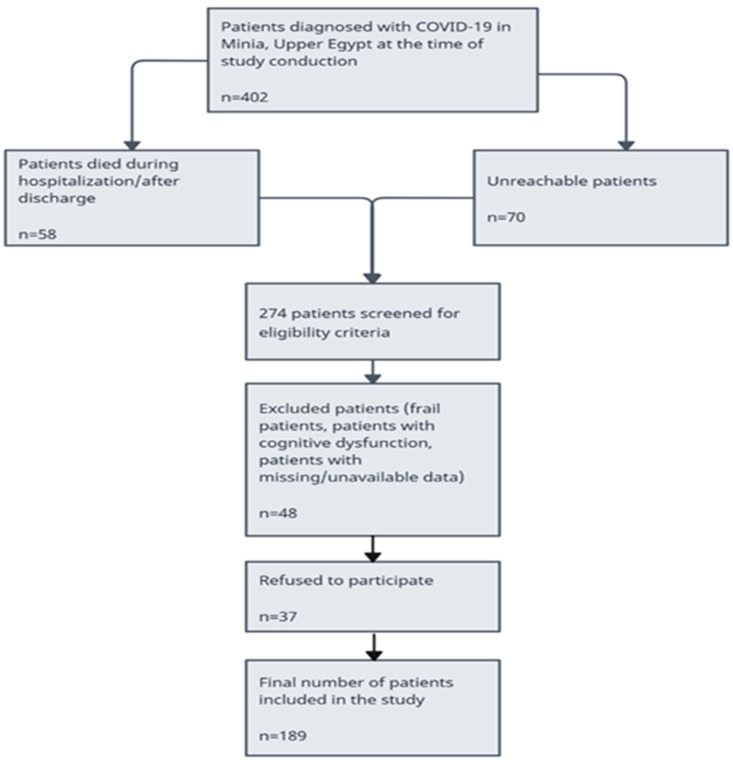
Flow chart for patients included in this study.

A total of 402 patients confirmed with COVID-19 were recruited to Minia University Hospitals. A total of 128 (31.8%) patients were not eligible for reasons shown in the participant’s flowchart (58 patients died during hospitalization or after discharge and 70 patients were unreachable) (Figure 1). A total of 48 patients were excluded because of cognitive dysfunction and unavailable data. A total of 226 patients were eligible for this study and invited to participate either in person or via telephone. A total of 189 patients agreed to participate and were included in this study analysis. Among patients studied, the median age was 38 years, and the distribution between genders was 31.2% men and 68.8% women. Among 56 patients (29.7%), the most prevalent comorbid condition was hypertension and diabetes mellitus followed closely. The number of patients hospitalized was 94 (49.7%), and the number of patients quarantined at home was 95 (50.3%). Six months after the initial COVID-19 diagnosis, 158 (83.5%) reported at least one persistent symptom. Fatigue was the most reported symptom, affecting 139 (73.5%) patients, followed by dyspnea, myalgia, and gastrointestinal problems. Descriptive statistics were used to summarize the prevalence of the different persistent symptoms among COVID-19 survivors (Table 2).

Sleep quality, exhaustion, coughing, and subjective dyspnea were evaluated using an eleven-point rating system ranging from 0 to 10. Symptom severity was categorized as mild (1–3), moderate (scores 4–6), or severe (scores 7–10). The mean and standard deviation (SD) of the symptoms and the distribution of symptom severity among individuals are reported in Table 3 and Table 4, respectively.

During this study, we performed chi-square tests to examine the relationship between medications used by patients and the incidence of developing persistent symptoms. The categories of drugs used were analgesics, antibiotics, corticosteroids, anticoagulants, and antivirals. There was no significant association found between the use of these different drugs and the development of long COVID (Table 5).

According to the Egyptian COVID-19 treatment protocol at the time of study conduction, ordinary people who are not at risk for developing a secondary bacterial infection or patients who had mild symptoms did not require the use of antibiotics. In our interviews, 21 patients with mild symptoms and 20 patients with moderate symptoms who were quarantined at home (did not require hospitalization and were not at risk of developing bacterial infections or sepsis) reported the self-administration of antibiotics (Table 6).

Simple logistic regression analysis revealed that having moderate symptom severity during the acute phase of the illness increased the probability of developing persistent COVID-19 symptoms later compared to having mild symptoms (OR 5.743, 95% CI 2.3205–14.21, *p* = 0.001). The analysis also revealed that increasing age was significantly associated with higher odds of developing persistent COVID-19 symptoms (OR = 1.028, 95% CI: 1.003–1.054, *p* = 0.030) (Table 7).

## 4. Discussion

The long-term sequelae of COVID-19 have represented a great issue for a while and remain a challenge for the foreseeable future. Many definitions for the condition have been suggested [16,17]. Because self-medication is especially common in low-income countries [8,10], we believed that the long-term effects of COVID-19 in Minia, Upper Egypt, were greatly underreported. In this study, we aimed to understand the degree to which these persistent symptoms have affected COVID-19 survivors and assess the incidence and severity of these symptoms after a minimum of 12 weeks of acute infection and their association with concurrent comorbidities and drug history in 189 survivors in Minia, Egypt. The symptoms described by survivors were examined six months after the acute phase of infection, which is consistent with the World Health Organization’s (WHO) definition of protracted COVID-19 as symptoms that persist or emerge more than 12 weeks after infection [18].

While long COVID has been widely studied, there remains a scarcity of data from low- and middle-income countries (LMICs), particularly from Upper Egypt. Our study contributes to the understanding of long COVID in a population where healthcare access, treatment approaches, and socioeconomic factors may influence symptom persistence differently than in high-income settings.

A total of 157 individuals (83.6%) reported at least one symptom after an average of 24 weeks from symptom onset. Fatigue (73.5%), myalgia (45.5%), as well as dyspnea (43.3%) were the most reported symptoms by participants. This is consistent with several studies, including a study completed in Spain and another one in Italy [19,20]. Persistent fatigue is described as a feeling of the physical loss of energy and increased difficulty performing particular tasks [21], and, according to the recent literature, it is one of the most reported persistent symptoms, regardless of initial illness severity [22,23,24]. According to the systematic review conducted by Jian et al., several factors could contribute to the development of persistent fatigue following infection with SARS-CoV-2, including biological or physical dysfunctions and the cytokines released during the infection [25].

Long COVID-19 does not only include symptoms related to the respiratory system or general constitutional symptoms. The term ‘long COVID-19’ is used to express a multi-organ disease, as many patients experienced neurological symptoms, either alone or in conjunction with pulmonary, cardiovascular, renal, dermatological, gastrointestinal, psychiatric, or immunological symptoms [26]. A recent large retrospective cohort study performed on 236,379 patients located primarily in the USA indicates that neuropsychiatric manifestations occurred in about a third of patients 6 months following the initial diagnosis [27]. Our analysis showed that new-onset persistent or fluctuating headaches and sleep disturbances were the most prevalent neurological symptoms among participants, followed by feelings of depression, anxiety, or distress. These findings are in line with a meta-analysis of 36 studies, which indicated that neurological symptoms affected about one-third of the pooled sample size [28]. Many theories attempted to explain these manifestations, including structural brain abnormalities and brainstem dysfunction [29,30]. Anxiety and stress during the acute phase of the illness and in the post-acute phase could lead to changes in neurotransmitters and vascular changes in the brain. SARS-CoV-2 can damage cerebral vasculature, leading to impaired oxygen and nutrient delivery to the brain, potentially explaining symptoms like dizziness and memory impairment [31]. Autoantibodies created in response to lingering viral components have also been thought to trigger inflammatory cells such as cytokines. Persistent immune activation, cytokine release (e.g., IL-6, TNF-α), and microglial activation have been linked to neuroinflammation, which may contribute to fatigue, headaches, and cognitive impairment [32,33,34]. Although direct invasion of the central nervous system (CNS) by SARS-CoV-2 is still debated, studies suggest viral persistence in neural tissues, which may play a role in post-COVID-19 neurological symptoms [34]. Post-viral dysautonomia, including postural orthostatic tachycardia syndrome (POTS), has been reported in long COVID patients, leading to symptoms such as palpitations, dizziness, and exercise intolerance [35]. In a meta-analysis published in 2021 that included 28 peer-reviewed studies and 7 preprints, involving 28,438 COVID-19 survivors, post-COVID-19 headache prevalence was 47.1% and decreased to 8.4% ≥180 days after onset/hospital discharge [36]. In another 9-month follow-up study of 905 patients, approximately 20% of patients had chronic headaches that were present on a daily basis [37]. This suggests that, while headache persistence varies, a significant subset of COVID-19 survivors continues to experience long-term headache symptoms. However, research in this area is still scarce, and the link between COVID-19 infection and developing new onset headaches needs to be explored further.

There are many potential mechanisms that may elucidate persistent symptoms. Long COVID is a multifactorial condition, with several proposed mechanisms contributing to the persistence of symptoms. Persistent immune activation and dysregulated inflammatory responses have been implicated in long COVID, potentially leading to prolonged fatigue, myalgia, and neurological symptoms [38]. Studies suggest that SARS-CoV-2 infection can induce endothelial injury and microvascular abnormalities, contributing to symptoms such as dyspnea, cognitive impairment, and cardiovascular manifestations [39,40]. The presence of viral reservoirs or molecular mimicry leading to autoimmune responses may explain the prolonged symptoms in some individuals [41]. Dysregulation of the autonomic nervous system and neuroinflammatory processes may play a role in post-COVID-19 fatigue, brain fog, and cardiovascular abnormalities [42].

Furthermore, there was an important association between moderate symptom severity during illness and developing persistent symptoms following recovery in comparison to individuals who were minimally symptomatic at the time of diagnosis, which concurs with the findings of a study conducted in Nigeria [43]. In a recent 2-year follow-up study in Finland, it was also shown that severe symptom severity at the time of infection corresponded to higher chances of having lingering symptoms [44]. When examining the impact of care setting, we noticed that hospitalization was associated with a higher chance of developing persistent symptoms, which is in agreement with a large cohort study conducted in Spain [45]. Another systematic review and meta-analysis of over 800,000 patients showed that being previously hospitalized or admitted to the ICU was associated with higher risks of developing PCC (OR, 2.37; 95% CI, 2.18–2.56) [45,46]. On the other hand, this outcome did not align with findings from a recent study based in Greece in which hospitalized and non-hospitalized patients had similar symptom patterns with no statistical significance with the exception of some symptoms, as there was a significant association between hospitalization and developing psychological and olfactory symptoms [47]. Differences in hospital resources, oxygen availability, medication access, or monitoring capabilities between the two healthcare systems might have influenced patient outcomes. When examining these results, it is important to note whether hospitalization is an independent factor of predicting long COVID, or if it is merely a proxy for initial symptom severity as patients with more severe symptoms are more likely to be hospitalized or admitted to the ICU.

This variation in findings could be justified by the fact that long COVID-19 symptoms are underreported in the population of this study, as most patients in our region choose to self-medicate instead of seeking medical advice from a physician [10]. Although the incidence of long COVID is higher in hospitalized compared to non-hospitalized patients, the incidence of long COVID is still relatively high in those who were not hospitalized (77.6%), and this could be explained by the possibility that some home-isolated patients had poor adherence to medications or suffered efficacy dosage regimen issues (low dose, correct dose but inappropriate frequency, short duration, or wrong timing) resulting in a failure of therapy, which may have played a role in the persistence of their symptoms. Also, these findings could be attributed to the fact that patients in this demographic have different behaviors and higher self-medication rates [8,10]. Patients who self-medicate might experience temporary symptom relief without addressing the underlying pathology, potentially masking acute symptoms while allowing inflammatory processes to continue, manifesting later as long COVID-19. While self-medication is a potential concern, other factors—such as healthcare access, disease severity, and pre-existing conditions—may also contribute to persistent symptoms.

The current findings also showed that a significant number of the patients did not require antibiotics in their treatment according to the Egyptian COVID-19 treatment protocol at the time of study conduction [48], including mildly symptomatic patients, who used antibiotics without a prescription. This underlines the phenomenon of self-medication in Upper Egypt and the irrational use of antibiotics [49,50]. This is especially problematic as many antibiotics could be obtained without a prescription in our region. During the interviews, some patients reported that they started using an antibiotic and then stopped it after one or two days because of unpleasant side effects, which could potentially lead to bacterial resistance, and the worsening of their disease had they developed a secondary bacterial infection at any point. This emphasizes the need to increase the awareness of this population about the nature and treatments of viral diseases as well as the proper use of antibiotics and bacterial resistance. Given the high prevalence of self-medication reported in this study, targeted public health interventions are urgently needed. Community education campaigns, stricter pharmacy regulations, and improved access to healthcare services could play a crucial role in reducing antibiotic misuse and its long-term consequences. Several studies highlighted that increasing knowledge, awareness, and practice among Egyptian populations decreased the incidence of misuse and prescribing antibiotics unnecessarily [51,52].

As the number of COVID-19 patients and ‘long-haulers’ increases, it is now increasingly important to continue investigating the impact of long-term COVID-19 on the daily lives of millions of people. About 1 million individuals in the UK and almost 18 million adults with COVID-19 in the United States are suffering from long COVID-19 symptoms [53,54,55]. Crucially, our findings indicate that the number of patients, both hospitalized and non-hospitalized, reporting persistent long COVID-19 symptoms after recovery is quite high (83.6%), which highlights the crucial role of proper follow-up following the recovery from COVID-19 in Minia, Upper Egypt. Although many studies and reviews have represented the problem of COVID-19 worldwide, studies are still scarce in this demographic. This is the first study of its kind to be applied in Minia, Upper Egypt. Furthermore, the unique characteristics of this population with its lower levels of awareness and approach to self-medication highlights the importance of studying this population with scrutiny.

There were some limitations to this study. The sample size is considered small com-pared to the total number of diagnosed and undiagnosed patients in Minia, Upper Egypt, because of the limitations in testing and follow-up capabilities. Recall bias was present in this study as participants were asked to retrospectively report self-medication and previous symptoms, which may have influenced some responses. The symptoms reported by the patients were subjective with no use of objective parameters such as pulmonary function tests, radiological, or laboratory testing. The lack of serological or PCR testing at the 6-month follow-up could have helped differentiate between ongoing infection and post-acute sequelae. Furthermore, the adherence of home-isolated patients to their medication regimen could not be assessed. Psychosocial and environmental factors such as anxiety, stress, and socioeconomic factors may contribute to symptom persistence, especially in low-resource settings. Some reported symptoms may be exacerbations of pre-existing comorbidities rather than direct consequences of COVID-19.

## 5. Conclusions

Long COVID-19 has been an increasingly prevalent concern in the medical community for the last four years and is still a burden on the healthcare system worldwide. Constitutional and neurological manifestations are two of the most persistent symptoms. The lack of appropriate levels of awareness about long COVID-19, as well as the tendency of patients in this demographic to self-medicate are leading causes of the underreporting of long COVID-19 symptoms in this region. Proper follow-up and identification of the factors predisposing to long-term COVID-19 are of major interest in Upper Egypt, as well as encouraging patients to reach out to healthcare providers whenever needed to avoid the complications of self-medication. Future research should explore targeted educational programs on antibiotic misuse in Upper Egypt.

## 6. Recommendations

Future studies should focus on the objective measures of symptom persistence, such as inflammatory biomarkers and diagnostic imaging along with patient-reported symptoms. This multi-method approach would not only validate self-reported outcomes but also shed light on how cultural norms, healthcare-seeking habits, and socioeconomic factors specific to this region may influence disease development and recovery patterns in widely prevalent illnesses.

Furthermore, large-scale, multicentric, prospective studies with a comprehensive methodology are essential to fully understand the complexities of long COVID.

It is also necessary to implement structured follow-up protocols post-recovery for patients and raise their awareness about appropriate thresholds for seeking medical attention. More importantly, future research should explore targeted educational programs on antibiotic misuse in Upper Egypt.

Future research should also incorporate more comprehensive methodologies, including prospective follow-ups, and assessments of health awareness and immune response.

## Figures and Tables

**Table 1:** Clinical characteristics of patients.

Patient Characteristic	N = 189	(%)
Age [in years]		
≤35	88	46.5%
>35–49	33	17.4%
50 and above	68	35.9%
Gender		
Male	59	31.2%
Female	130	68.8%
Comorbidity		
Present	56	29.6%
Absent	133	70.4%
Smoking		
Yes	42	22.2%
No	147	77.8%
Number of Comorbidities		
No comorbidities	133	70.4%
1 comorbid condition	41	21.7%
2 or more comorbidities	15	17.9%
Comorbidity		
Hypertension	36	19%
DM	16	8.4%
Asthma	5	2.6%
Thyroid disease	2	1%
Stroke	1	0.52%
Others ^a^	3	1.5%
Place of Quarantine		
Hospital	94	49.7%
Home	95	50.3%
Initial COVID-19 Severity		
Asymptomatic	4	2.1%
Mild	57	30.1%
Moderate	93	49.2%
Severe	35	18.5%

^a^ Other: Three patients had peptic ulcer disease (PUD).

**Table 2:** Self-reported persistent symptoms of COVID-19 survivors.

System	Symptom	N = 189 (%)
General	Fatigue	139 (73.5)
	Myalgia	86 (45.5)
	Extremity swelling	9 (4.7)
Respiratory	Dyspnea	82 (43.3)
	Cough	79 (41.7)
	Chest discomfort	48 (25.3)
Cardiovascular	Palpitations	4 (2.1)
Gastrointestinal	Appetite changes	25 (13.2)
	Abdominal discomfort	41 (21.6)
Neurological	Headache	57 (30.1)
	Confusion	17 (8.9)
	Anxiety	40 (21.1)
	Loss of taste	26 (13.7)
	Loss of smell	30 (15.8)
	Sexual dysfunction	1 (0.5)
	Sleep disturbance	64 (33.8)

**Table 3:** Mean and SD for shortness of breath (SOB), cough, fatigue, insomnia.

Symptom	Mean	SD
SOB	0.81	(±1.3)
Cough	0.88	(±1.3)
Fatigue	2.27	(±1.3)
Insomnia	1.02	(±1.3)

**Table 4:** Distribution of symptom severity.

	No Symptom (0)	Mild (1–3)	Moderate (4–6)	Severe (7–10)
SOB	110	67	11	1
Cough	106	77	4	2
Fatigue	47	101	30	11
Insomnia	121	54	5	9

**Table 5:** Chi-square for the comparison of drugs used during acute COVID-19 symptoms between patients with and without long COVID symptoms.

		Long COVID		*p* Value
		No	Yes	
		N = 31	N = 158	
Analgesics	No	2 (6.5%)	9 (5.7%)	0.870
	Yes	29 (93.5%)	149 (94.3%)	
Antibiotics	No	18 (58.1%)	64 (40.5%)	0.0713
	Yes	13 (41.9%)	94 (59.5%)	
Corticosteroids	No	27 (87.1%)	135 (85.4%)	0.810
	Yes	4 (12.9%)	23 (14.6%)	
Anticoagulants	No	29 (93.5%)	127 (80.4%)	0.077
	Yes	2 (6.5%)	31 (19.6%)	
Antivirals	No	30 (96.8%)	142 (89.9%)	0.219
	Yes	1 (3.2%)	16 (10.1%)	

**Table 6:** Patients who reported self-administration of antibiotics.

Patients with mild symptoms	21
Patients with moderate symptoms	20

**Table 7:** Simple logistic regression analysis for prediction of patients with long COVID-19 syndrome.

Predictor	Odds Ratio	95% Confidence Interval		*p* Value
		Lower	Upper	
Age	1.028	1.003	1.054	0.030 *
Symptom Severity				
Mild				
Moderate	5.743	2.3205	14.21	0.001 *
Hospitalization				
Yes	2.384	1.055	5.387	0.037 *

* *p* < 0.05.

## Data Availability

Data are contained within the article.
